# Supplementary material for: Fermentable Oligosaccharides, Disaccharides, Monosaccharides, and Polyols Reintroduction in Clinical Practice: Surveying the Gaps and Opportunities
Source: Gastro Hep Adv. 2026 Mar 5;5(5):100908. doi: 10.1016/j.gastha.2026.100908 (PMC13054409; doi:10.1016/j.gastha.2026.100908)
Supplement: Supplementary File [file mmc1.pdf]

Supplementary file
Reintroduction phase questionnaire

What is your practice setting?

a) Academic or University Hospital
b) Community Hospital
c) Private practice
d) Other _____________

How often do you start low FODMAP diet for your patients?

a) Multiple times a week
b) Once a week
c) Once every 2 weeks
d) Once a month

The following questions pertain to the **reintroduction phase** of low FODMAP diet. Please choose the most appropriate response for your practice.

1. Do you challenge patients with each FODMAP using:
   a) Single dose once a day (e.g. ¼ cup mushroom once daily)
   b) Single doses multiple times a day (e.g. ¼ cup mushroom at lunch and another ¼ cup at dinner)
   c) Single doses once a day but in increasing amount (e.g. ¼ cup mushroom once daily on day 1 and 1/3 cup mushroom once daily on day 2 and so on)
   d) Single doses multiple times a day but in increasing amount (e.g. ¼ cup mushroom twice daily on day 1 and if tolerated 1/3 cup mushroom twice daily on day 2 and so on)
   e) Varying doses in same day (1/4 cup of mushroom for lunch and 1/3rd cup for dinner)
   f) None of the above

2. The starting dose of each FODMAP challenge for the majority of patients is:
   a) How much a patient eats of that particular FODMAP
   b) A standard dose set by you/your group/literature

3. For each FODMAP group, how many food items do you generally challenge patients with before moving on the next FODMAP challenge:
   a) One
   b) Two
   c) Three
   d) More than three

4. When teaching patients about FODMAP reintroduction, do you usually meet patients:
   a) One on one
   b) Group session
   c) Both

5. For most of your patients, as they go through the reintroduction process, how often do you meet them:
   a) One visit
   b) Two visits
   c) Three visits
   d) More than three visits

6. For the majority of patients, the sequence of which FODMAP challenge to test first is decided by:
   a) Patient
   b) Dietitian
   c) Shared decision between the patient and the dietitian

7. If the sequence of FODMAP challenge is decided by/have input from the dietitian, how do you usually decide on the sequence of FODMAP challenge:
   a) Based on symptoms
   b) Based on individual FODMAP consumption (after diet recall)
   c) Others ___________________________

8. For the majority of the patients, do you ask patients to reintroduce a FODMAP group at a ***particular dose (e.g. ½ mango) without increasing over***
   a) 1 day; recommend increasing dose after.
   b) 2-3 days
   c) 4-6 days
   d) 7 days or more

9. For the majority of the patients, the sequence of **increasing dose amount** of FODMAP challenge is decided by:
   a) Patient
   b) Dietitian
   c) Shared decision between the patient and the dietitian

10. For the majority of the patients, do you ask patients to **complete a particular FODMAP reintroduction (e.g. polyol)** including any increase in dose over
    a) 2-3 days
    b) 4-6 days
    c) 7-10 days
    d) 11-15 days

11. For the majority of the patients, how long do you wait between challenges with FODMAP groups (e.g. lactose and fructans, fructans and polyols, etc.) if they **did not** react to the previous FODMAP group
    a. 1-3 days
    b. 4-6 days
    c. 7 days or more

12. For the majority of the patients, how long do you wait between challenges with FODMAP groups (e.g. fructans and galactans, galactans and polyols, etc.) if they **did** react to the previous FODMAP group?
    a. 1-3 days

b. 4-6 days
c. 7 days or more
d. Varies by patient depending on severity of symptoms

13. How long does the **entire** reintroduction process take for most of your patients?

a. Less than a month
b. 1-2 months
c. 3-4 months
d. 5-6 months
e. >6 months

14. Do you give handouts to patients for the reintroduction phase?

f. Yes
g. No

15. Do you always or most of the time meet your patients for additional visit(s) after they finish the reintroduction phase

a. Yes
b. No
