# Supplementary material for: Fermentable Oligosaccharides, Disaccharides, Monosaccharides, and Polyols Reintroduction in Clinical Practice: Surveying the Gaps and Opportunities
Source: Gastro Hep Adv. 2026 Mar 5;5(5):100908. doi: 10.1016/j.gastha.2026.100908 (PMC13054409; doi:10.1016/j.gastha.2026.100908)
Supplement: Extended PDF [file mmc2.pdf]

Gastro Hep Advances 2026;5:100908

ORIGINAL RESEARCH—CLINICAL

Fermentable Oligosaccharides, Disaccharides, Monosaccharides, and Polyols Reintroduction in Clinical Practice: Surveying the Gaps and Opportunities

Kate Pelletier,[1] Megan Villarreal,[1] Rowan Klar,[2] William D. Chey,[1] Prashant Singh,[1] Allen Lee,[1] Yen-Po Wang,[1,3] and Amanda Lynett[1]

[1]Division of Gastroenterology and Hepatology, University of Michigan Health System, Ann Arbor, Michigan; [2]Department of Umich School of Public Health, Nutritional Sciences, University of Michigan School of Public Health, Ann Arbor, Michigan; [3]Division of Endoscopy Diagnosis and Treatment, Department of Internal Medicine, Taipei Veterans General Hospital, Taipei, Taiwan

BACKGROUND AND AIMS: The low fermentable oligosaccharides, disaccharides, monosaccharides, and polyols diet (LFD) consists of 3 phases: restriction, reintroduction, and personalization. Despite the importance of the reintroduction phase, there are limited real-world data on its implementation. This study examined variations in registered dietitians' (RDs) practices during LFD reintroduction. **METHODS:** Cross-sectional national online surveys were conducted. One hundred forty-five RDs were recruited via professional networks, social media, and listservs. Survey questions assessed food dosage at initiation, progression, challenge doses, number of foods tested per subtype, and reintroduction duration. **RESULTS:** Participants worked in private practice (50%) and academic hospitals (26%). Over half of RDs challenge 1 food per fermentable oligosaccharides, disaccharides, monosaccharides, and polyols group, while 37% use 2 or more. Reintroduction sequencing is typically collaborative (73%), though some RDs leave it to patients. In the absence of symptoms, most wait 1–3 days before increasing doses, while 20% wait over 4 days. When symptoms occur, challenge timing is adjusted based on severity, though 37% follow fixed intervals. Nearly, all RDs (98%) conduct reintroductions one-on-one, with 63% completing them within 1–2 months and 37% taking longer. While the reintroduction practices patterns were similar between academic and nonacademic settings, academic RDs met with patients less frequently during reintroduction phase but more likely to meet with patients after reintroduction compared to nonacademic RDs ($P < .05$ for both). **CONCLUSION:** The variability in LFD reintroduction practices underscores the need for a standardized protocol. While most dietitians offer one-on-one sessions and follow-up care, inconsistencies in timing, frequency, and sequencing persist. Although shared decision-making is common, approaches to managing reactions and determining challenge timing vary, highlighting the need for clearer guidelines.

Keywords: Irritable Bowel Syndrome; Low FODMAP Diet; Reintroduction Phase; Dietitians

Introduction

Irritable bowel syndrome (IBS) is a disorder of gut-brain interaction characterized by symptoms of recurrent abdominal pain, associated with abnormal bowel habits.[1] IBS affects 4%–10% of the global population and is associated with a significant negative impact on quality of life and work productivity along with direct costs related to health-care utilization.[2,3] A diet low in fermentable oligosaccharides, disaccharides, monosaccharides, and polyols (FODMAPs) (LFD) is the most evidence-based dietary intervention for managing IBS. More recently, the LFD has been shown to be superior to medical therapy in IBS and proposed as first-line therapy for IBS.[4,5]

The current approach to LFD is a 3-step process starting with a restriction phase followed by reintroduction and personalization phases. In the restriction phase, high-FODMAP foods are eliminated from the diet for a period (typically 4–6 weeks) to assess symptom improvement.[6] Following this, the reintroduction phase consists of reintroducing FODMAP-containing foods to identify potential triggers. Finally, the personalization phase uses information from the reintroduction phase to tailor a long-term diet that minimizes symptom-inducing foods while maintaining dietary variety. Registered dietitians (RDs) are essential throughout this process, providing education, monitoring, and ensuring that nutritional needs are met despite the dietary restrictions.[7,8]

While the restriction phase has been well studied and standardized, the reintroduction phase lacks widely accepted standards, leading to inconsistent clinical practices.[9] This phase is inherently more complex and nuanced, and current research provides limited guidance on best practices.[10] This lack of standardization has led to variability in how the reintroduction phase is conducted in

Abbreviations used in this paper: **FODMAPs, fermentable oligosaccharides, disaccharides, monosaccharides, and polyols; IBS, irritable bowel syndrome; LFD, low FODMAP diet; RDs, registered dietitians.**

Most current article

© 2026 The Author(s). Published by Elsevier Inc. on behalf of American Gastroenterological Association Institute. This is an open access article under the CC BY license (http://creativecommons.org/licenses/by/4.0/).
2772-5723
https://doi.org/10.1016/j.gastha.2026.100908

both research and real-world practice. As a result, patients may receive inconsistent guidance during this stage of the LFD. This is highlighted in a recent survey where >40% of patients reported this phase as difficult to implement.[11]

To date, there has been no survey of dietitians regarding their practice patterns with regard to the reintroduction phase of the LFD. Given these gaps, the present study aims to explore how RDs approach the reintroduction phase in real-world clinical practice. By examining these practices, we hope to (1) describe real-world practice patterns with regard to the reintroduction phase and understand if there were differences in reintroduction practices with respect to practice settings and (2) identify areas of education, standardization, and research related to the reintroduction phase.

Materials and Methods

This was a cross-sectional survey study of RDs working in various settings including academic institutions, community hospitals, private practices, outpatient clinics, retail, telehealth, college campuses, Program of All-Inclusive Care for the Elderly sites, and primary care. The survey was developed on Qualtrics with input from 2 gastroenterologists and 4 RDs. It consisted of 16 questions using a mix of Likert scale and multiple-choice formats (Supplementary File).

The survey was distributed electronically via professional networks, email, social media, and listservs—including the Academy of Nutrition and Dietetics' Dietitians in Gluten and Gastrointestinal Disorders group, the Michigan Academy of Nutrition and Dietetics, the International Foundation for Gastrointestinal Disorders, University of Michigan outpatient RDs, and the FOOD: The Main Course to Digestive Health Conference and its Instagram account. Informed consent was implied by voluntary survey completion. Responses were collected anonymously over 4 months, yielding a total of 208 responses for analysis.

Survey Instrument

A structured questionnaire was developed to capture detailed information on the approaches RDs engage in for LFD initiation, FODMAP challenge protocols, patient education strategies, and follow-up practices. The survey included both closed-ended and multiple-choice questions and covered the following domains: practice setting (academic/university, private practice, community hospital/other), frequency of LFD initiation, dosing strategies for FODMAP challenges, number of food items used per FODMAP group, patient education format (one-on-one, group, or both), frequency and number of follow-up visits, decision-making processes for FODMAP reintroduction, duration and sequencing of challenges, and use of educational handouts and postreintroduction follow-up (Supplementary File).

Statistical Analysis

Descriptive statistics were used to summarize the overall responses (Table 1). Frequencies and percentages were calculated for categorical variables. Comparative analyses were conducted to examine differences in LFD practices across practice settings (academic/university, private practice, and community hospital/other), as shown in Table 2. Chi-square tests were used to assess statistical significance, with a P value of <.05 considered significant.

Results

A total of 145 RDs responded to the survey. The majority of respondents identified as delivering care in private practice (n = 72, 49.7%), followed by academic/university (n = 38, 26.2%) or community hospital/other settings (n = 35, 24.1%) (Table 1).

Variability in LFD Reintroduction

Dose of FODMAP challenges. There was considerable heterogeneity in the delivery of LFD reintroduction, including the starting dose and the overall dose of FODMAP challenges. While 117 (80.7%) utilized a standard dose set by themselves, their group, and/or the literature, 28 (19.3%) used the amount of each particular FODMAP typically consumed by the patient as the starting dose. While the majority (71.7%) of dietitians reported using escalating once-daily doses for reintroduction, 19.3% prefer a consistent once-daily dose throughout the challenge, and the remaining 8.9% use multiple daily doses or other dosing methods, as outlined in Table 1.

Number of challenges per FODMAP group. The number of items per each FODMAP group to challenge patients with before moving on to the next FODMAP challenge was also variable, with 91 respondents (63.2%) reporting 1 item, 26 (18.1%) reporting 2 items, 21 (14.5%) reporting 3 items, and 6 (4.2%) reporting more than 3 items (Figure 1A).

Duration of the challenge and intervals between challenges. When there was no reaction to the challenge, the length of time to complete a particular FODMAP reintroduction was variable, with 97 respondents (68.3%) recommending 2–3 days, 30 (21.2%) recommending 4–6 days, 11 (7.7%) recommending 7–10 days, and 4 (2.8%) recommending 11–15 days per challenge (Figure 1B). Most RDs (79.9%) would wait 1–3 days before proceeding to the next FODMAP challenge when there was no reaction (Figure 1C).

When there was a reaction to a FODMAP challenge, the majority of the RDs (90, 62.5%) would wait depending on an individual patient's symptom severity. However, others would wait a fixed interval as highlighted in Table 1 and Figure 1D. Over half of the RDs (63.1%) reported completing the entire FODMAP reintroduction process within 2 months (Figure 1E).

Number of visits. The number of visits for patients as they go through the reintroduction process was also varied among RDs, with 37 respondents (26.1%) recommending 1 visit, 47 (33.1%) 2 visits, 26 (18.3%) 3 visits, and 32 (22.5%) recommending more than 3 visits.

Table 1. Results of Reintroduction Phase Questionnaire of Registered Dietitians

Question	Response	N	%
Practice pattern	Academic/university	38	26.2
	Private practice	72	49.7
	Community hospital/other	35	24.1
Frequency of LFD start	Multiple times a week	47	32.4
	Once a week	25	17.2
	Once every 2 wk	25	17.2
	Once every month	48	33.1
Dose of FODMAP challenge	Single doses once a day but in increasing amounts	104	71.7
	Single doses multiple times a day but in increasing amounts	5	3.4
	Single doses multiple times a day	2	1.4
	Single dose once a day	28	19.3
	None of the above	6	4.1
Starting dose for each FODMAP challenge	A standard dose set by you/your group/ literature	117	80.7
	How much a patient eats of that particular FODMAP	28	19.3
For each FODMAP group, how many items do you generally challenge patients with before moving on to the next FODMAP challenge?	1	91	63.2
	2	26	18.1
	3	21	14.5
	More than 3	6	4.2
When teaching patients about FODMAP reintroduction, do you usually meet patients?	One-on-one	142	97.9
	Group session	1	0.7
	Both	2	1.4
For most of your patients, as they go through the reintroduction process, how often do you meet them?	1 visit	37	26.1
	2 visits	47	33.1
	3 visits	26	18.3
	More than 3 visits	32	22.5
For the majority of patients, the sequence of which FODMAP challenge to test first is decided by	Patient	37	25.7
	Dietitian	2	1.4
	Shared patient-dietitian decision	105	72.9
For the majority of patients, do you ask patients to reintroduce a FODMAP group at a particular dose without increasing over?	1 d (ie, recommend increasing dose after 1 d)	115	79.7
	2–3 d	20	13.9
	4–6 d	1	0.7
	7 d or more	4	2.8
	I do not generally recommend increasing the dose	4	2.8
For the majority of patients, increasing dose amount of FODMAP challenge (for each FODMAP group) is decided by	Patient	15	10.4
	Dietitian	25	17.4
	Shared patient-dietitian decision	101	70.1
	N/A as I do not recommend increasing the dose	3	2.1
For the majority of patients, do you ask patients to complete a particular FODMAP reintroduction (eg, polyol) including any increase dose over?	2–3 d	97	68.3

Table 1. Continued

Question	Response	N	%
	4–6 d	30	21.2
	7–10 d	11	7.7
	11–15 d	4	2.8
For the majority of patients, how long do you wait between challenges with FODMAP groups (eg, lactose and fructans, fructans and polyols, etc) if they did not react to the previous FODMAP group?	1–3 d	115	79.9
	4–6 d	24	16.7
	7 d or more	5	3.5
For the majority of patients, how long do you wait between challenges with FODMAP groups (eg, fructans and galactans, galactans and polyols, etc) if they did react to the previous FODMAP group?	1–3 d	19	13.2
	4–6 d	26	18.1
	7 d or more	9	6.3
	Varies by patient depending on severity of symptoms	90	62.5
How long does the entire reintroduction process take for most of your patients?	Less than 1 mo	5	3.4
	1–2 mo	86	59.7
	3–4 mo	46	31.9
	5–6 mo	4	2.8
	More than 6 mo	3	2.1
Do you give handouts to patients for the reintroduction phase?	No	10	6.9
	Yes	119	82.1
	Maybe	16	11.0
Do you always or most of the time meet your patients for additional visit(s) after they finish the reintroduction phase?	No	29	20.0
	Yes	116	80.0

FODMAP, fermentable oligosaccharides, disaccharides, monosaccharides, and polyols; LFD, low FODMAP diet.

Variability in LFD Reintroduction by Practice

There was significant variability in practice patterns between RDs in academic/university, private, or community hospital practices related to LFD reintroduction. Dietitians in academic/university practices met less frequently during the reintroduction phase but more likely to meet after finishing the reintroduction phase than those in a nonacademic settings ($P = .047$ and .021, respectively) (Figure 2A and B). Furthermore, academic RDs were more likely to use a fixed starting dose (decided by literature or RD) for FODMAP challenges and took less time to complete a particular FODMAP reintroduction compared to nonacademic dietitians ($P = .049$ and .042, respectively) (Figure 2C and D) (Table 2). The length of time to complete a particular FODMAP reintroduction was also different between groups ($P = .042$) (Figure 2E). There were no differences between the groups with regard to other parameters studied (Table 2).

Discussion

This study highlights considerable variability in how RDs implement the reintroduction phase of the LFD in clinical practice. Despite the existence of general guidance and expert consensus for this phase,[12] our findings reveal a lack of standardization across multiple aspects of the process, including FODMAP challenge dosing, food selection, timing between challenges, and overall duration of this phase. These differences appeared across all practice settings, with statistically significant variation identified in key clinical decisions such as starting dose selection, visit frequency, and reintroduction timing.

We also found that there was significant variation and overall lack of consensus on main components, such as the number of items tested per FODMAP subgroup, starting dose and dose escalation for reintroduction, timing of challenge, and interval between the challenges. This raises concerns about consistency in patient education and outcome

Table 2. Results of Reintroduction Phase Questionnaire of Registered Dietitians Categorized by Practice Patterns Version 2

		Practice setting			
Questions	Answers	Academic/ university N (%)	Private practice N (%)	Community hospital/other N (%)	*P* value
How often do you start LFD?	Multiple times a week	18 (47.4)	22 (30.6)	7 (20.0)	.080
	Once a week	5 (13.2)	16 (22.2)	4 (11.4)	
	Once every 2 wk	3 (7.9)	14 (19.4)	8 (22.9)	
	Once every month	12 (31.6)	20 (27.8)	16 (45.7)	
Dose of FODMAP challenge	Single doses once a day but in increasing amounts	29 (76.3)	50 (69.4)	25 (71.4)	.929
	Single doses multiple times a day but in increasing amounts	0 (0)	4 (5.6)	1 (2.9)	
	Single doses multiple times a day	0 (0)	1 (1.4)	1 (2.9)	
	Single dose once a day	7 (18.4)	14 (19.4)	7 (20.0)	
	None of the above	2 (5.3)	3 (4.2)	1 (2.9)	
Starting dose for each FODMAP challenge	A standard dose set by you/ your group/literature	32 (84.2)	62 (86.1)	23 (65.7)	.049
	How much a patient eats of that particular FODMAP	6 (15.8)	10 (13.9)	12 (34.3)	
For each FODMAP group, how many items do you generally challenge patients with before moving on to the next FODMAP challenge?	25 (65.8)	46 (63.9)	20 (58.8)	25 (65.8)	.735
	5 (13.2)	15 (20.8)	6 (17.6)	5 (13.2)	
	5 (13.2)	9 (12.5)	7 (20.6)	5 (13.2)	
	3 (7.9)	2 (2.8)	1 (2.9)	3 (7.9)	
When teaching patients about FODMAP reintroduction, do you usually meet patients?	One-on-one	35 (92.1)	72 (100)	35 (100)	.062
	Group session	1 (2.6)	0 (0)	0 (0)	
	Both	2 (5.3)	0 (0)	0 (0)	
For most of your patients, as they go through the reintroduction process, how often do you meet them?	1 visit	16 (42.1)	10 (14.1)	11 (33.3)	.047
	2 visits	12 (31.6)	25 (35.2)	10 (30.3)	
	3 visits	5 (13.2)	15 (21.1)	6 (18.2)	
	More than 3 visits	5 (13.2)	21 (29.6)	6 (18.2)	
For the majority of patients, the sequence of which FODMAP challenge to test first is decided by	Patient	6 (15.8)	20 (28.2)	11 (30.6)	.350
	Dietitian	0 (0)	2 (2.8)	0 (0)	
	Shared patient-dietitian decision	32 (84.2)	49 (69.0)	25 (69.4)	
For the majority of patients, do you ask patients to reintroduce a FODMAP group at a particular dose without increasing over?	1 d (ie, recommend increasing dose after 1 d)	32 (84.2)	56 (77.8)	27 (77.1)	.730
	2–3 d	4 (10.5)	11 (15.3)	5 (14.3)	
	4–6 d	0 (0)	1 (1.4)	1 (2.9)	
	7 d or more	2 (5.3)	2 (2.8)	0 (0)	
	I do not generally recommend increasing the dose	0 (0)	2 (2.8)	2 (5.7)	

Table 2. Continued

		Practice setting			
Questions	Answers	Academic/ university N (%)	Private practice N (%)	Community hospital/other N (%)	*P* value
For the majority of patients, increasing dose amount of FODMAP challenge (for each FODMAP group) is decided by	Patient	3 (7.9)	5 (6.9)	7 (20.6)	.022
	Dietitian	7 (18.4)	17 (23.6)	1 (2.9)	
	Shared patient-dietitian decision	28 (73.7)	49 (68.1)	24 (70.6)	
	N/A as I do not recommend increasing dose	0 (0)	1 (1.4)	2 (5.9)	
For the majority of patients, do you ask patients to complete a particular FODMAP reintroduction (eg, polyol) including any increase in dose over?	2–3 d	27 (71.1)	45 (63.4)	25 (75.8)	.042
	4–6 d	4 (10.5)	21 (29.6)	5 (15.2)	
	7–10 d	6 (15.8)	2 (2.8)	3 (9.1)	
	11–15 d	1 (2.6)	3 (4.2)	0 (0)	
For the majority of patients, how long do you wait between challenges with FODMAP groups (eg, lactose and fructans, fructans and polyols, etc) if they did not react to the previous FODMAP group?	1–3 d	34 (89.5)	55 (77.5)	26 (74.3)	.089
	4–6 d	2 (5.3)	13 (18.3)	9 (25.7)	
	7 d or more	2 (5.3)	3 (4.2)	0 (0)	
For the majority of patients, how long do you wait between challenges with FODMAP groups (eg, fructans and galactans, galactans and polyols, etc) if they did react to the previous FODMAP group?	1–3 d	4 (10.5)	10 (13.9)	5 (14.7)	.719
	4–6 d	4 (10.5)	16 (22.2)	6 (17.6)	
	7 d or more	2 (5.3)	4 (5.6)	3 (8.8)	
	Varies by patient depending on severity of symptoms	28 (73.7)	42 (58.3)	20 (58.8)	
How long does the entire reintroduction process take for most of your patients?	<1 mo	0 (0)	1 (1.4)	4 (11.8)	.277
	1–2 mo	23 (60.5)	43 (59.7)	20 (58.8)	
	3–4 mo	13 (34.2)	24 (33.3)	9 (26.5)	
	5–6 mo	1 (2.6)	3 (4.2)	0 (0)	
	>6 mo	1 (2.6)	1 (1.4)	1 (2.9)	
Do you give handouts to patients for the reintroduction phase?	No	5 (13.2)	4 (5.6)	1 (2.9)	.358
	Yes	30 (78.9)	61 (84.7)	28 (80.0)	
	Maybe	3 (7.9)	7 (9.7)	6 (17.1)	
Do you always or most of the time meet your patients for additional visit(s) after they finish the reintroduction phase?	No	5 (13.2)	11 (15.3)	13 (37.1)	.021
	Yes	33 (86.8)	61 (84.7)	22 (62.9)	

FODMAP, fermentable oligosaccharides, disaccharides, monosaccharides, and polyols; LFD, low FODMAP diet.

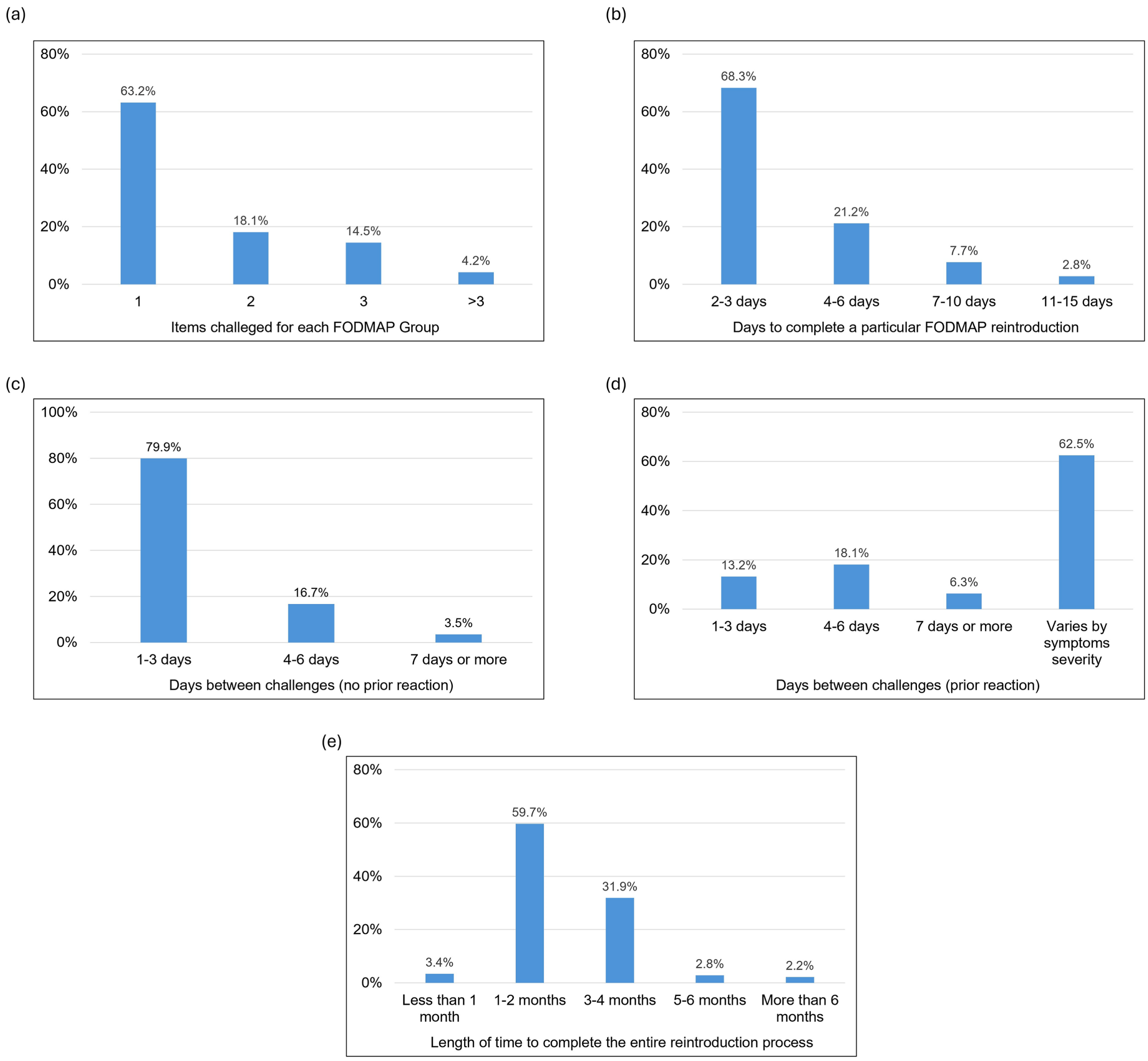

Figure 1. Results of reintroduction phase questionnaire of registered dietitians (RDs). (A) The numbers of items in each fermentable oligosaccharides, disaccharides, monosaccharides, and polyols (FODMAP) group RDs generally rechallenged patients with before next challenge. (B) The time RDs ask patients to complete a particular FODMAP reintroduction. (C) The time the RDs wait between FODMAP groups challenges if patients did not react to previous FODMAP group. (D) The time the RDs wait between FODMAP groups challenges if patients did not react to previous FODMAP group. (E) The entire length of time for patients to complete the entire reintroduction process.

assessments. For example, although the expert recommendation is to finish a particular challenge in 2–3 days,[13] almost one-third of RDs challenge over a longer duration as the most common method in their practice. Furthermore, more than a third of RDs answered that the reintroduction process takes longer than 2 months for their patients to complete. Similar heterogeneity was observed in the starting dose, increase in dose (if any) of challenges, and number of food items tested in each FODMAP group. All these factors can significantly impact patient outcomes (whether they consider themselves sensitive to FODMAP group vs not), the duration of reintroduction phase, and their ease/difficulty in navigating this phase.

The heterogeneity observed in the conduct of FODMAP reintroduction highlights the individualized nature of the LFD, but it also signals a potential gap between existing recommendations and real-world applications. While personalization is essential due to patient-specific symptoms and tolerances, the absence of more detailed, standardized protocols may lead to inconsistent care among providers, patient confusion on identifying specific FODMAP intolerances,[6] and variable outcomes. For example, nearly half of the respondents (43.3%) reported adjusting the wait time between FODMAP challenges based on symptom severity of a reaction, which is indicative of personalized nature of this phase. However, a considerable proportion of RDs do not adopt this recommendation as the most common approach to dealing with symptom reproduction with challenges.

There were significant differences by practice setting, particularly in the determination of starting doses ($P = .049$), frequency of RD visits during LFD reintroduction education ($P = .047$), and reintroduction duration ($P = .042$), suggesting that institutional factors may

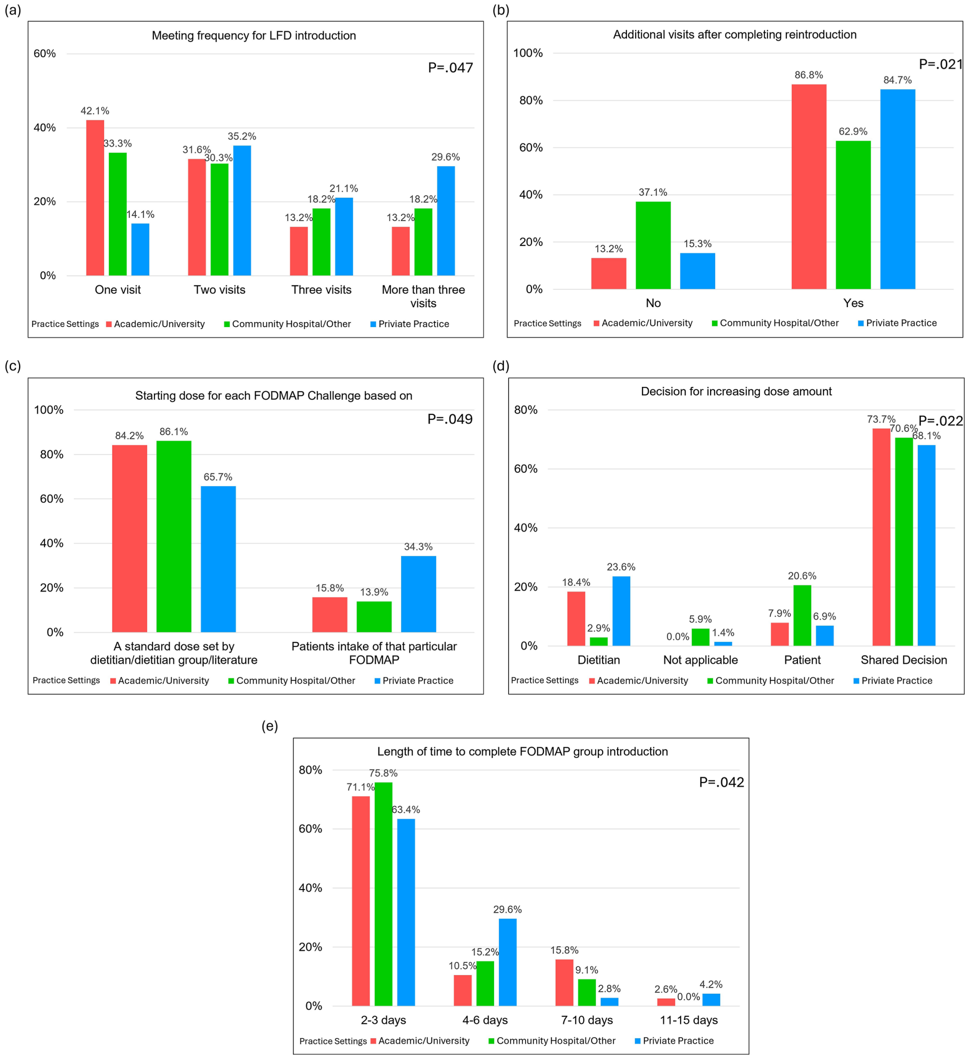

Figure 2. The difference in fermentable oligosaccharides, disaccharides, monosaccharides, and polyols (FODMAP) reintroduction among practice patterns of registered dietitians (RDs). (A) The meeting times of RDs takes for reintroduction process. (B) The need of additional visits after FODMAP reintroduction. (C) The decision of starting dose for each FODMAP challenge. (D) The decision mode on increasing amount of FODMAP challenge. (E) The time duration RDs let patients to complete a particular FODMAP reintroduction.

influence clinical decision-making. Private practice RDs, for instance, may have more flexibility in scheduling and follow-up than those working in hospital settings, which could impact how the reintroduction phase is implemented. These differences could reflect varying access to resources, training, or patient populations and may contribute to different patient experiences depending on the care setting.

These findings reinforce the need for improved standardization and clearer guidance on how to structure the reintroduction phase of the LFD, as suggested by prior studies.[10] They also highlight several opportunities for research and education. First, it is possible that significant variation in reintroduction practices largely stems from lack of research in this phase. The few research studies in this phase have largely focused on identifying the most common triggers[14,15] but not comparing different reintroduction strategies. Second, until more data become available, there remains a need for expert consensus to develop best practices around FODMAP reintroduction. Third, more education and training around the reintroduction and personalization phases will hopefully reduce variability while maintaining the personalized aspects of these phases of the LFD.

While we found significant variability in the above-mentioned aspects of RD practices, we also found areas of greater consistency—for example, more than 80% of RDs gave handouts to patients and also met with them once they had completed the reintroduction process. This highlights the sustained support provided by RDs for patients as they transition into the personalization phase of the LFD, which is important to ensure greater diet quality and diversity while maintaining symptom improvement.[12]

This study had several limitations. First, data were self-reported, which may introduce bias in how respondents described their practices. Second, although the sample was national in scope, it may not be fully representative of all RDs who implement the LFD, particularly those working outside of gastrointestinal nutrition, given that the survey was shared mostly through gastrointestinal nutrition platforms. Additionally, the survey's fixed-choice design may not have captured the full nuance of clinical decision-making, such as why specific practices were chosen or how RDs handle complex patient presentations. Further details on specific questions, such as types of handouts provided to patients for the LFD reintroduction phase,

rather than learning if they provided handouts or not, could be useful information when further forming more standardized approaches.

Conclusions

This study demonstrates considerable variability in how RDs implement the reintroduction phase of the LFD in clinical practice. Notable differences were observed in dosing strategies, food selection, reintroduction timelines, and follow-up practices. These findings reveal inconsistencies while emphasizing the need for evidence-based, standardized protocols to ensure consistent patient care and improve clinical outcomes. As the reintroduction phase remains poorly studied, establishing clear protocols and expert consensus would enhance clinical decision-making and support ongoing patient management. Future research should focus on refining these practices and addressing the gaps in training to provide more uniform, effective guidance for RDs across diverse clinical settings.

Supplementary Materials

Material associated with this article can be found, in the online version, at https://doi.org/10.1016/j.gastha.2026.100908.

References

1. Lacy BE, Mearin F, Chang L, et al. Bowel disorders. Gastroenterology 2016;150:1393–1407.e5.
2. Ford AC, Sperber AD, Corsetti M, et al. Irritable bowel syndrome. Lancet 2020;396:1675–1688.
3. Buono JL, Carson RT, Flores NM. Health-related quality of life, work productivity, and indirect costs among patients with irritable bowel syndrome with diarrhea. Health Qual Life Outcomes 2017;15:35.
4. Carbone F, Van den Houte K, Besard L, et al. Diet or medication in primary care patients with IBS: the DOMINO study - a randomised trial supported by the Belgian Health Care Knowledge Centre (KCE Trials Programme) and the Rome Foundation Research Institute. Gut 2022;71:2226–2232.
5. Nybacka S, Törnblom H, Josefsson A, et al. A low FODMAP diet plus traditional dietary advice versus a low-carbohydrate diet versus pharmacological treatment in irritable bowel syndrome (CARIBS): a single-centre, single-blind, randomised controlled trial. Lancet Gastroenterol Hepatol 2024;9:507–520.
6. Whelan K, Martin LD, Staudacher HM, et al. The low FODMAP diet in the management of irritable bowel syndrome: an evidence-based review of FODMAP restriction, reintroduction and personalisation in clinical practice. J Hum Nutr Diet 2018;31:239–255.
7. Chey WD, Hashash JG, Manning L, et al. AGA clinical practice update on the role of diet in irritable bowel syndrome: expert review. Gastroenterology 2022;162:1737–1745.e5.
8. Scarlata K, Eswaran S, Baker JR, et al. Utilization of dietitians in the management of irritable bowel syndrome by members of the American College of Gastroenterology. Am J Gastroenterol 2022;117:923–926.
9. Black CJ, Staudacher HM, Ford AC. Efficacy of a low FODMAP diet in irritable bowel syndrome: systematic review and network meta-analysis. Gut 2022;71:1117–1126.
10. Tuck C, Barrett J. Re-challenging FODMAPs: the low FODMAP diet phase two. J Gastroenterol Hepatol 2017;32(Suppl 1):11–15.
11. Fehlman J, Peng W, Pfennig C, et al. Tu1982: low FODMAP expounded: a new elucidation on patient attitudes, challenges and outcomes. Gastroenterology 2025;169:S-1582.
12. Staudacher HM, Whelan K. The low FODMAP diet: recent advances in understanding its mechanisms and efficacy in IBS. Gut 2017;66:1517–1527.
13. Rhys-Jones D. Reintroduction using the diary function. MONASH university. August 5, 2022. Accessed September 1, 2025. https://www.monashfodmap.com/blog/reintroduction%2Dusing%2Ddiary%2Dfunction/
14. Van den Houte K, Colomier E, Routhiaux K, et al. Efficacy and findings of a blinded randomized reintroduction phase for the low FODMAP diet in irritable bowel syndrome. Gastroenterology 2024;167:333–342.
15. Eswaran S, Jencks KJ, Singh P, et al. All FODMAPs aren't created equal: results of a randomized reintroduction trial in patients with irritable bowel syndrome. Clin Gastroenterol Hepatol 2025;23:351–358.e5.

Received September 29, 2025. Accepted February 25, 2026.

Correspondence:
Address correspondence to: Yen-Po Wang, MD, PhD, Division of Gastroenterology and Hepatology, University of Michigan Health System, University of Michigan, 1500 East Medical Center Drive, 3912 Taubman Center, Ann Arbor, Michigan 48109. e-mail: yenpow@umich.edu or ulnafu@gmail.com.

Authors' Contributions:
Kate Pelletier: General design of the study; data acquisition; drafting of the article. Megan Villarreal: General design of the study; data acquisition; interpretation of data; drafting of the article. Rowan Klar: General design of the study; data analysis; interpretation of data. William D. Chey: General design of the study; data analysis; critical revision of the article. Prashant Singh: General design of the study; data acquisition; data analysis; drafting of the article; critical revision of the article. Allen Lee: General design of the study; data acquisition; data analysis; interpretation of data; drafting of the article; critical revision of the article. Yen-po Wang: Data acquisition; data analysis; interpretation of data; drafting of the article. Amanda Lynett: General design of the study; data acquisition; data analysis; interpretation of data; drafting of the article; critical revision of the article.

Conflicts of Interest:
These authors disclose the following: William D. Chey: Consultant: Ardelyx, Atmo, Gemelli, Phathom, Redhill, Salix, and Vibrant; Research Support: Salix; Stock Options: Evinature, Food Marble, Kiwi Bioscience, and Modify Health; Patents: Digital Manometry, Rectal Expulsion Device. Allen Lee has consulted for GlaxoSmithKline and Atmo Biosciences. The remaining authors disclose no conflicts.

Funding:
A. Lee is supported by grants from The Leona M. and Harry B. Helmsley Charitable Trust and the National Institutes of Health grants DK124567 and DK139095.

Ethical Statement:
The study was determined to be exempt from institutional review board review (HUM#00269351). The project involved only surveys and did not collect protected health information or other identifiable information. Data were recorded anonymously, and participants could not be readily identified. As such, the study met criteria for research involving minimal risk.

Data Transparency Statement:
The data that support the findings of this study are available from the corresponding author upon reasonable request.

Reporting Guidelines:
Reporting Guidelines were not applicable for this article type.

Gastro Hep Advances, Volume 5

Supplemental information

Fermentable Oligosaccharides, Disaccharides, Monosaccharides, and Polyols Reintroduction in Clinical Practice: Surveying the Gaps and Opportunities

Kate Pelletier, Megan Villarreal, Rowan Klar, William D. Chey, Prashant Singh, Allen Lee, Yen-Po Wang, and Amanda Lynett

Supplementary file
Reintroduction phase questionnaire

What is your practice setting?

a) Academic or University Hospital
b) Community Hospital
c) Private practice
d) Other _____________

How often do you start low FODMAP diet for your patients?

a) Multiple times a week
b) Once a week
c) Once every 2 weeks
d) Once a month

The following questions pertain to the **reintroduction phase** of low FODMAP diet. Please choose the most appropriate response for your practice.

1. Do you challenge patients with each FODMAP using:
   a) Single dose once a day (e.g. ¼ cup mushroom once daily)
   b) Single doses multiple times a day (e.g. ¼ cup mushroom at lunch and another ¼ cup at dinner)
   c) Single doses once a day but in increasing amount (e.g. ¼ cup mushroom once daily on day 1 and 1/3 cup mushroom once daily on day 2 and so on)
   d) Single doses multiple times a day but in increasing amount (e.g. ¼ cup mushroom twice daily on day 1 and if tolerated 1/3 cup mushroom twice daily on day 2 and so on)
   e) Varying doses in same day (1/4 cup of mushroom for lunch and 1/3rd cup for dinner)
   f) None of the above

2. The starting dose of each FODMAP challenge for the majority of patients is:
   a) How much a patient eats of that particular FODMAP
   b) A standard dose set by you/your group/literature

3. For each FODMAP group, how many food items do you generally challenge patients with before moving on the next FODMAP challenge:
   a) One
   b) Two
   c) Three
   d) More than three

4. When teaching patients about FODMAP reintroduction, do you usually meet patients:
   a) One on one
   b) Group session
   c) Both

5. For most of your patients, as they go through the reintroduction process, how often do you meet them:
   a) One visit
   b) Two visits
   c) Three visits
   d) More than three visits

6. For the majority of patients, the sequence of which FODMAP challenge to test first is decided by:
   a) Patient
   b) Dietitian
   c) Shared decision between the patient and the dietitian

7. If the sequence of FODMAP challenge is decided by/have input from the dietitian, how do you usually decide on the sequence of FODMAP challenge:
   a) Based on symptoms
   b) Based on individual FODMAP consumption (after diet recall)
   c) Others ___________________________

8. For the majority of the patients, do you ask patients to reintroduce a FODMAP group at a ***particular dose (e.g. ½ mango) without increasing over***
   a) 1 day; recommend increasing dose after.
   b) 2-3 days
   c) 4-6 days
   d) 7 days or more

9. For the majority of the patients, the sequence of **increasing dose amount** of FODMAP challenge is decided by:
   a) Patient
   b) Dietitian
   c) Shared decision between the patient and the dietitian

10. For the majority of the patients, do you ask patients to **complete a particular FODMAP reintroduction (e.g. polyol)** including any increase in dose over
    a) 2-3 days
    b) 4-6 days
    c) 7-10 days
    d) 11-15 days

11. For the majority of the patients, how long do you wait between challenges with FODMAP groups (e.g. lactose and fructans, fructans and polyols, etc.) if they **did not** react to the previous FODMAP group
    a. 1-3 days
    b. 4-6 days
    c. 7 days or more

12. For the majority of the patients, how long do you wait between challenges with FODMAP groups (e.g. fructans and galactans, galactans and polyols, etc.) if they **did** react to the previous FODMAP group?
    a. 1-3 days

b. 4-6 days
c. 7 days or more
d. Varies by patient depending on severity of symptoms

13. How long does the **entire** reintroduction process take for most of your patients?
a. Less than a month
b. 1-2 months
c. 3-4 months
d. 5-6 months
e. >6 months

14. Do you give handouts to patients for the reintroduction phase?
f. Yes
g. No

15. Do you always or most of the time meet your patients for additional visit(s) after they finish the reintroduction phase
a. Yes
b. No
